# Comparison of the acute phase protein and antioxidant responses in dogs vaccinated against canine monocytic ehrlichiosis and naive-challenged dogs

**DOI:** 10.1186/s13071-015-0798-1

**Published:** 2015-03-23

**Authors:** Nir Rudoler, Shimon Harrus, Silvia Martinez-Subiela, Asta Tvarijonaviciute, Michael van Straten, Jose J Cerón, Gad Baneth

**Affiliations:** Koret School of Veterinary Medicine, Hebrew University, P.O. Box 12, Rehovot, 76100 Israel; Interdisciplinary Laboratory of Clinical Pathology, Interlab-UMU, Campus of Excellence Mare Nostrum, University of Murcia, 30100 Espinardo, Murcia Spain

**Keywords:** *Ehrlichia canis*, 611A strain, Vaccination, Acute phase proteins

## Abstract

**Background:**

Canine monocytic ehrlichiosis (CME) is a tick-borne disease with a global distribution, caused by *Ehrlichia canis*. The inflammatory response to *E. canis* infection includes changes in certain acute phase proteins (APP) and in biomarkers of the oxidative status. APP responses are considered part of the innate immune response to CME. The aim of this study was to evaluate the APP and oxidative marker responses in dogs vaccinated against CME with an attenuated vaccine and subsequently challenged with a wild *E. canis* strain.

**Methods:**

The study included 3 groups of 4 beagle dogs. Group 1 dogs were inoculated subcutaneously with an attenuated *E. canis* vaccine on day 0, and again on day 213. Group 2 initially served as controls for group 1 during the vaccination phase and then vaccinated once on day 213. Group 3 consisted of naïve dogs which constituted the control group for the challenge phase. All 12 dogs were infected intravenously with a wild strain of *E. canis* on day 428 of the study. APP levels were serially measured during two periods: days 0–38 post-vaccination (groups 1 and 2) and days 0–39 post-challenge (groups 1, 2, 3).

**Results:**

Changes in C-reactive protein (CRP), serum amyloid A (SAA), haptoglobin, albumin, paraoxonase-1 (PON-1) and total antioxidant capacity (TAC) were of significantly smaller magnitude in vaccinated dogs and appeared later on a time scale compared to unvaccinated dogs challenged with a wild strain. Alterations in the level of APP during the vaccination phase of the study were of lower extent compared to those in the challenged unvaccinated dogs during the post-challenge phase. Positive APP levels correlated positively with the rickettsial load, body temperature and negatively with the thrombocyte counts (p < 0.05).

**Conclusions:**

Vaccination with an attenuated *E. canis* strain and challenge with a wild strain resulted in considerably reduced responses of positive and negative APP, and oxidative biomarker responses in vaccinated compared to unvaccinated dogs, reflecting a milder innate inflammatory response conferred by protection of the vaccine.

**Electronic supplementary material:**

The online version of this article (doi:10.1186/s13071-015-0798-1) contains supplementary material, which is available to authorized users.

## Background

Canine monocytic ehrlichiosis (CME) is an important canine disease of worldwide distribution. The etiological agent of CME is the obligate intracellular rickettsia *Ehrlichia canis*, a tick-borne bacterium that causes a multisystemic disease that typically induces severe bleeding tendencies due to thrombocytopenia and thrombocytopathy. The acute disease is characterized by high fever, depression, lethargy, anorexia, lymphadenomegaly, splenomegaly, and hemorrhages. Acutely infected dogs may recover or remain infected sub-clinically and eventually develop chronic CME. Dogs that develop the chronic form of the disease suffer from bone marrow suppression and decreased hematopoiesis with clinical signs similar to those in the acute phase with a greater severity [1,2].

The pathogenesis of CME which includes the induction of immune-mediated phenomena such as immune complex formation, anti-thrombocyte antibodies and bone marrow suppression remains to be elucidated [1-3]. One important aspect of the acute phase of CME is the alteration in the production of certain plasma proteins including the acute phase proteins (APP) that participate in the inflammatory response to *E. canis* infection [4,5]. The APP are considered to be non-specific innate immune components involved in the restoration of homeostasis and restraint of microbial growth before the host develops acquired immunity to an external challenge [6,7]. They consist of “positive” and “negative” proteins that show an increase or decrease in level, respectively, after an inflammatory stimulus.

The positive APP include C-reactive protein (CRP) which plays important roles in protection against infection, clearance of damaged tissue, prevention of auto-immunization and regulation of the inflammatory response [8]. Other positive APP include haptoglobin (Hp) that binds free hemoglobin, inhibits its oxidative activity and antagonizes its pro-inflammatory activity [9], and serum amyloid A (SAA) whose roles include detoxification of endotoxins, inhibition of lymphocyte and endothelial cell proliferation, inhibition of platelet aggregation, and inhibition of T-lymphocyte adhesion to extracellular matrix proteins [10]. The negative APP include albumin, the most abundant constitutive plasma protein, which serves as a source of nutrients and regulator of osmotic pressure [11]. Anti-oxidants grouped under the term Total Antioxidant Capacity (TAC) defend cells and tissues from harmful oxidative damage [12] whereas paraoxonase-1 (PON-1) is an important enzyme involved in lipid metabolism which is down-regulated during oxidative stress [13].

The main objective of this study was to evaluate the APP and oxidative responses in dogs vaccinated against CME with a live attenuated vaccine and subsequently challenged with a wild isolate of the bacterium, and compare them to the responses in non-vaccinated dogs challenged with infection. In addition, correlations between the APP and oxidative responses, and the hematological changes and blood bacterial loads were evaluated. As the APP and oxidative responses have rarely been studied in response to vaccination in dogs, the association between these responses and the protection conferred by vaccination during challenge is of major interest.

## Methods

### Experimental infection of dogs with *Ehrlichia canis*

Twelve laboratory-bred, 12–24 months old female beagle dogs were used in this study as previously described in a publication on the efficacy of this vaccine [14]. The dogs were acclimatized for 4–5 weeks before the initiation of the study and divided into 3 groups of 4 dogs each (Table 1). Group 1 dogs were initially inoculated subcutaneously (SQ) with 4.8×10^9^ attenuated *E. canis* strain 611A bacteria (the vaccine strain) on day 0, and again on day 213 with 9.6×10^9^ of the *E. canis* vaccine strain bacteria SQ. Group 2 dogs were initially inoculated SQ with ~1.2×10^6^ uninfected DH-82 cells (day 0) as control for group 1 and then with 9.6×10^9^ vaccine strain bacteria on day 213. The third group (group 3) consisted of naïve dogs which constituted the control group for the challenge study, when both groups 1 and 2 were vaccinated. They joined the study on day 393, were acclimatized for 12 days, and were then subcutaneously inoculated with ~1.2×10^6^ uninfected DH-82 cells on day 405. Twenty three days later (day 428), all 12 dogs were intravenously inoculated with 6 ml *E. canis* infected blood containing 6×10^7^*E. canis* wild strain bacteria, drawn from a clinically acute ill dog [14]. Quantitation of rickettsial load both in the cultures and the blood was determined by quantitative real-time PCR (qPCR) as described below. The *E. canis* infected blood was tested microscopically by stained blood smear examination and no other blood pathogens could be detected. It was also screened molecularly for *Hepatozoon canis* DNA using primers Hep-F and Hep-R [15], for *Babesia* spp. DNA using the Piro-A and Piro-B primers [16], and in addition with primers 107F and 299R targeted at the *ompA* gene fragment for spotted fever rickettsiae [17]. The *E. canis* infected blood was negative by all these PCR assays.

**Table 1:** **The different groups of dogs and their function during the two phases of the study**

**Study phase**	**Group number**	**Group treatment**	**Group function in respective phase**
Post-vaccinal	1	Vaccinated once with vaccine strain	Evaluation of response to vaccination
	2	Inoculated with uninfected DH-82 cells	Control for group 1
Post-challenge	1	Vaccinated twice with vaccine strain and challenged with virulent *E. canis* strain	Evaluation of protection by vaccine and response to challenge
	2	Vaccinated once with vaccine strain and challenged with virulent *E. canis* strain	Evaluation of protection by vaccine and response to challenge
	3	Inoculated with uninfected DH-82 cells and challenged with virulent *E. canis* strain	Control for groups 1 and 2

Monitoring of the dogs included daily inspection, physical examination at least twice weekly and a weekly bodyweight recording. Five ml of blood were drawn from each dog in EDTA and serum tubes at least once weekly and complete blood count analysis was carried out using the ADVIA 120® Hematology system (Bayer, Germany). Serum was separated from whole blood and frozen at −80°C until used for APP and oxidative biomarkers measurements.

The study was carried out according to the Hebrew University guidelines for animal experimentation and was approved by the Institutional Animal Care and Use Committee (approval numbers MD-09-11937-4 and MD-12869-4).

### Treatment

Azithromycin (Azithromycin, 200 mg/5 ml, Teva, Israel) treatment was administered to all 4 dogs in group 3 in an effort to test for its efficacy in CME. It was initiated on day 15 post-challenge (day 443) when all four dogs presented fever, anorexia, lethargy and thrombocytopenia. The planned treatment protocol was 7 mg/kg, PO q 24 hrs for 5 days as a loading dose followed by the same dose q 72 hrs for additional 15 days [18]. The initial loading dose was administered for 4 days. However, it was discontinued due to severe clinical deterioration of 2 of the 4 dogs with no improvement of any treated dog. At this stage (day 447), since azithromycin was not found to be effective it was replaced by doxycycline (10 mg/kg, PO, once daily) which was administered for an additional 21 days [14]. No medical treatment was needed for the vaccinated dogs in groups 1 and 2.

### DNA extraction

DNA was extracted using a commercial kit (Illustra blood genomicPrep mini spin kit, GE health care, UK), following the manufacturer’s instructions.

### Quantitative real-time PCR

A quantitative estimation of the *E. canis* rickettsial load was performed by qPCR using the Rotor-Gene 6000 Real-Time PCR analyzer (Corbett life sciences, Australia) and the *E. canis*-16S plasmid as previously described [19]. Standard curve was designed using decimal dilutions of the *E. canis*-16S plasmid. The primers used to target the *16S rRNA* gene were the *E. canis* 16S forward TCGCTATTAGATGAGCCTACGT and the *E. canis* 16S reverse GAGTCTGGACCGTATCTCAGT [14].

### Acute phase protein measurement

The concentration of CRP was determined with a solid sandwich immunoassay (Tridelta Phase range canine CRP kit; Tridelta development Ltd., Bray, Ireland). The final absorbance of the samples was measured in a microtitre plate at 450 nm using 630 nm as the reference (Powerwave XS, Biotek instruments, Carson City, NV, USA). Hp was determined using a hemoglobin-binding method (Tridelta phase, Tridelta Development Ltd., Bray Ireland) in a biochemistry autoanalyzer (Cobas Mira Plus, ABX Diagnostics, Montpellier, France). SAA concentration was measured using a sandwich immunoassay (Tridelta Phase range SAA assay, Tridelta development Ltd., Bray, Ireland). Final absorbance of the samples was measured by use of microtitre plate reader (Powerwave XS, Biotek instruments, Carson City, NV, USA). Albumin concentration was determined by using the bromocresol green-dye binding method using a commercial kit [20]. TAC was measured using a colorimetric method developed by Erel [21] and previously used in dogs by Camkerten and others [22] and Tvarijonaviciute and others [23]. Serum PON1 activity was determined measuring arylesterase activity with p-nitrophenol acetate as substrate following a previously described method for use in dogs [23].

PON, TAC and albumin were measured in serum on an automated biochemistry analyzer (Olympus AU600 Automatic Chemistry Analyzer, Olympus Europe GmbH, Hamburg, Germany). All APP have been previously validated in the reference laboratory and all samples were analyzed in the same analytical run to avoid high between-run imprecision as had been previously reported for CRP and SAA [24].

### Study periods

The study included two short phases in the experiment: the post-vaccination and challenge phases. The APP levels and the *E. canis* rickettsial loads analyzed in this study were measured and compared to hematologic findings from dogs in groups 1 and 2 during 38 days post-vaccination (post-vaccinal phase) when group 1 was vaccinated and group 2 served as its control; and 39 days post-challenge (challenge phase) when vaccinated groups 1 and 2 were compared to unvaccinated group 3 and to each other (Table 1). The post-vaccinal period corresponds to days 0 to 38 of the study, and the challenge phase from days 428 to 467 of the study.

### Statistical analysis

All analyses were done using SAS 9.3 software. *p* values <0.05 were considered statistically significant.

#### APP and oxidative marker levels

A marginal model was used for this analysis. Treatment group and sample day (i.e. time effect) entered the model as fixed effects, while repeated measurements within dogs were dealt with by adding a complex error term which included a correlation matrix to account for the repeated measurements of the same dog. The correlation matrix used was auto-regressive (AR(1)). The model we used was:

$$ \mathrm{Y} = \mathrm{T}\mathrm{ime}\ \left(6\ \mathrm{index}\ \mathrm{variables}\right) + \mathrm{T}\mathrm{R}\mathrm{T}\left(2\hbox{--} 3\mathrm{index}\ \mathrm{variables}\right) + \mathrm{T}\mathrm{ime}*\mathrm{T}\mathrm{R}\mathrm{T} + \mathrm{e}, $$

where Y is the analyzed APP, TRT- is treatment, and e is the complex error term representing the within-dog correlation of blood sample results and the residual error. The results reported are least squares means and are referred to in the model results section simply as means.

#### Correlation between APP and oxidative markers and clinical and hematological parameters

As the number of observations was small, correlation between APP and oxidative markers and clinical and hematological parameters was estimated using the Spearman correlation coefficient (*r*). Values of the coefficient vary between “0”, indicating no statistical dependence between two variables, and “1” indicating a total statistical dependence between 2 variables.

## Results

### Clinical and hematological findings

No clinical signs were observed in the post-vaccinal phase. Reduction in the number of thrombocytes was found to be the only hematological parameter that was altered during this phase. No clinical signs were observed among the vaccinated dogs. Following the challenge with the wild virulent *E. canis* strain, 3 dogs from the vaccinated groups developed transient mild to moderate fever. In addition, thrombocytopenia was detected among all vaccinated dogs and returned to normal reference range without therapeutic intervention. In contrast, the control dogs developed severe clinical signs including lethargy, anorexia and persistent fever. Furthermore, 2 of the control dogs developed life threatening clinical disease with severe hypothermia, lethargy and anorexia. The clinical signs and thrombocytopenia experienced by the control group were reversed only after initiation of doxycycline treatment.

### Serum levels of APP

The concentrations of all APP during the two study phases are shown in Additional file 1: Table S1. Table 2 presents the peak APP and oxidative marker concentrations and the ratios between peaks and baseline levels within the groups.

**Table 2:** **Changes (least square means values) in APP and oxidative markers and change rates detected during post-vaccination and post-challenge periods**

**Inflammatory marker**	**Phase of study**	**Group**	**Pre-vaccination or challenge value (standard error)**	**Highest value for positive APP and lowest value for negative APP (standard error)**	**Day of detection (post-vaccination or challenge)**	Rate of change*
**CRP (μg/mL)**	vaccination	1	6.9 (4.5)	70.6 (11.52)	24	10.23
		2	5 (0)	8.65 (10.66)	13	1.73
	challenge	1	5 (0)	112 (23.31)	22	22.4
		2	5 (0)	173 (23.31)	22	34.6
		3	5 (0)	252 (23.31)	14	50.4
**Hp (g/L)**	vaccination	1	1.68 (1.62)	5.04 (0.73)	24	3
		2	0.28 (0.23)	1.88 (0.69)	24	6.7
	challenge	1	1.56 (0.16)	4.86 (1.21)	22	3.11
		2	1.77 (1.17)	3.78 (1.05)	22	2.13
		3	2.13 (0.29)	8.60 (1.05)	14	4.03
**SAA (μg/mL)**	vaccination	1	5.1 (2.39)	23.94 (2.9)	24	4.69
		2	3.43 (1.92)	5.1 (2.5)	13	1.48
	challenge	1	1.43 (0.97)	61 (9.9)	22	42.65
		2	2.05 (1.29)	36 (8.6)	22	17.56
		3	1.66 (0.43)	156.77 (8.6)	14	94.43
**Albumin (g/dL)**	vaccination	1	3.07 (0.06)	2.33 (0.26)	31	0.75
		2	3 (0.31)	3.01 (0.29)	31	1
	challenge	1	3.64 (0.4)	2.8 (0.15)	22	0.76
		2	3.99 (0.24)	2.8 (0.15)	22	0.7
		3	3.79 (0.19)	1.64 (0.17)	22	0.43
**TAC (mmol/L)**	vaccination	1	0.36 (0.16)	0.07 (0.06)	31	0.19
		2	0.45 (0.017)	0.04 (0.06)	39	0.08
	challenge	1	0.36 (0.06)	0.12 (0.08)	22	0.33
		2	0.46 (0.1)	0.2 (0.06)	14	0.43
		3	0.24 (0.13)	0.08 (0.03)	14	0.33
**PON-1 (IU/mL)**	vaccination	1	2.91 (0.27)	2.1 (0.16)	31	0.72
		2	2.43 (0.29)	2.2 (0.16)	38	0.9
	challenge	1	3.64 (0.6)	2.1 (0.23)	22	0.57
		2	3.9 (0.68)	2.05 (0.23)	22	0.52
		3	3.62 (0.11)	1.3 (0.23)	22	0.35

*Highest or lowest least square means value divided by the mean pre-vaccination or pre-challenge value of the same group.

### Model results

#### CRP

##### Post-vaccination

On day 24 post-vaccination, CRP levels in group 1 were higher by 33.87 μg/mL than those in group 2 (*p* = 0.008). In groups 1 and 2, CRP levels were 37.62 (*p* = 0.005) and 57.03 (*p* = 0.005) μg/mL greater on days 12 and 24, respectively, when compared to pre-vaccination levels.

##### Post-challenge

**Figure 1:**
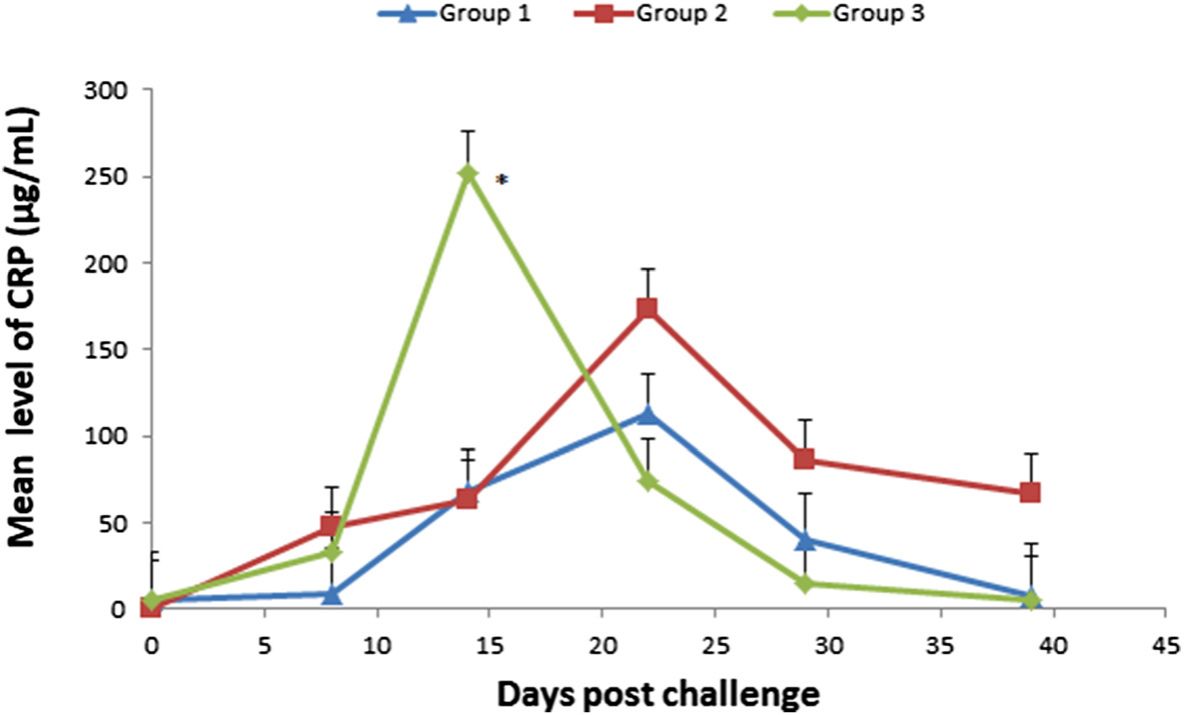
**CRP kinetics in vaccinated and unvaccinated dog groups during the post-challenge phase.** Least square means sera level of C-reactive protein (CRP) in two groups of vaccinated dogs (group 1 and 2) and a group of control unvaccinated dogs (group 3) following challenge with a wild strain of *E. canis*. *Indicates significant difference between group 3 and group 1 (*p* = 0.003).

Following challenge with the wild virulent strain, CRP levels on day 14 post-challenge in group 3 were 184.13 μg/mL greater than in group 1 (*p* = 0.003) (Figure 1). In all groups, CRP values were on average 107.32 μg/mL greater on day 22 post-challenge when compared to pre-challenge values (*p* = 0.002). No significant differences were detected between group 1 and group 2 during this phase.

#### Hp

##### Post-vaccination

No significant differences were found between groups 1 and 2 during the post-vaccination phase. Both groups had significantly higher mean Hp levels on day 14 and 22 post-vaccination compared to day 0 pre-vaccination (1.67 g/L, *p* = 0.050, and 2.83 g/L, *p* = 0.006, respectively).

##### Post-challenge

On day 14, Hp levels in group 3 were 6.12 g/L greater than in group 1 (*p* = 0.007). On day 22 post-challenge, mean Hp levels were 3.28 g/L greater in all groups, when compared to pre-challenge levels (*p* = 0.040). No differences between groups 1 and 2 were found.

#### SAA

##### Post-vaccination

Levels of SAA were 17.61 μg/mL greater in group 1 compared to group 2 on day 24 post-vaccination (*p* = 0.003). In both groups, mean SAA values were 18.77 μg/mL greater on day 24 post-vaccination compared to pre-vaccination values (*p* < 0.001).

##### Post-challenge

**Figure 2:**
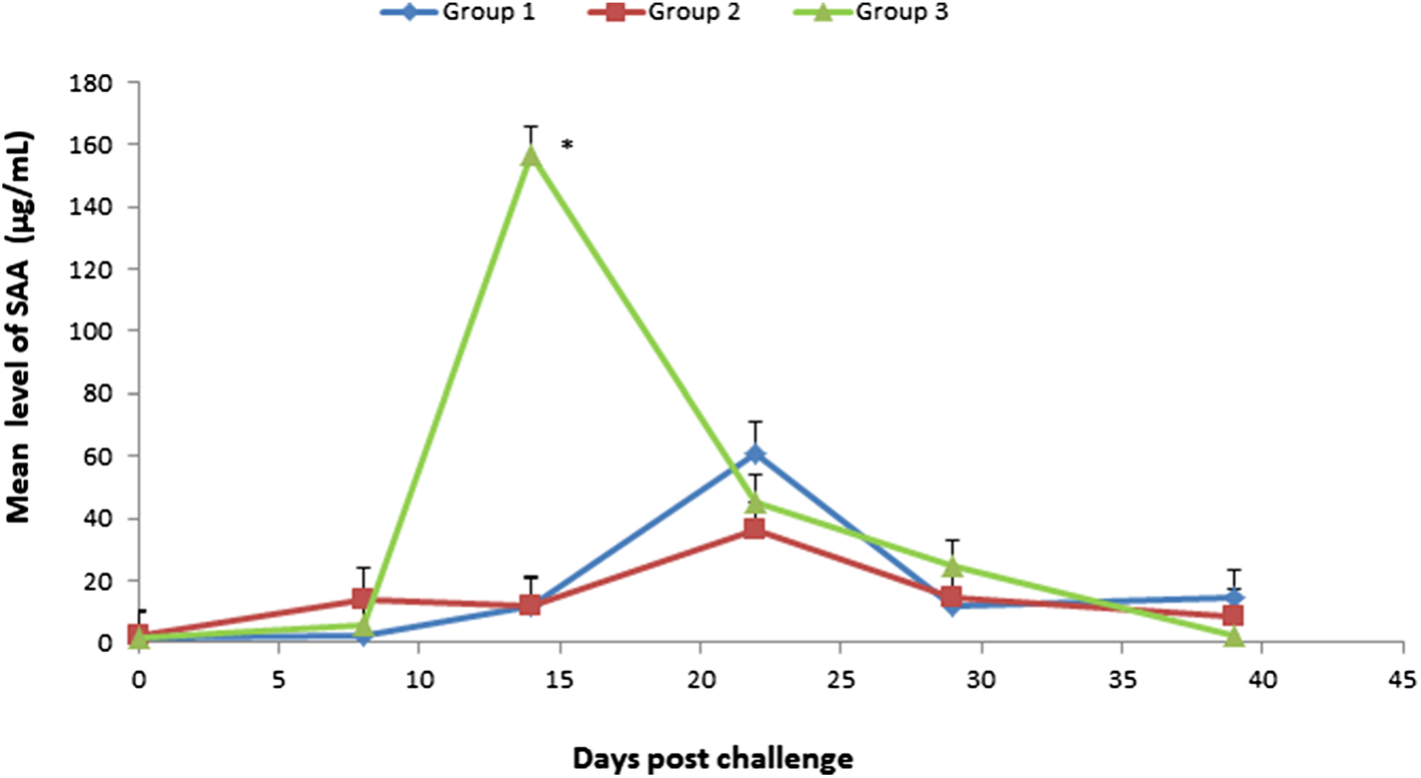
**SAA kinetics in vaccinated and unvaccinated dog groups during the post-challenge phase.** Least square means sera level of serum amyloid A (SAA) in vaccinated dogs (group 1 and 2) and control unvaccinated dogs (group 3) following challenge with a wild strain of *E. canis*. *Indicates significant difference between group 3 and group 1 (*p <* 0.001).

On day 14, SAA values in group 3 were 144.91 μg/mL greater than in group 1 (*p* < 0.001) (Figure 2). In addition, mean SAA levels on day 22 in all groups were 59.34 μg/mL greater than pre-challenge levels (*p* < 0.001). No differences between groups 1 and 2 were found.

#### Albumin

##### Post-vaccination

Mean albumin levels in group 2 were 1.42 g/dL greater on day 6 post-vaccination, when compared to day 0 (*p* = 0.01). Significantly lower mean levels of albumin were detected in groups 1 and 2 on day 6 post-vaccination. The mean reduction was by 1.14 g/dL (*p* = 0.01). A similar trend was observed on day 29 post-vaccination in which a mean reduction of 0.74 g/dL was detected (*p* = 0.05) compared to the levels prior to vaccination.

##### Post-challenge

**Figure 3:**
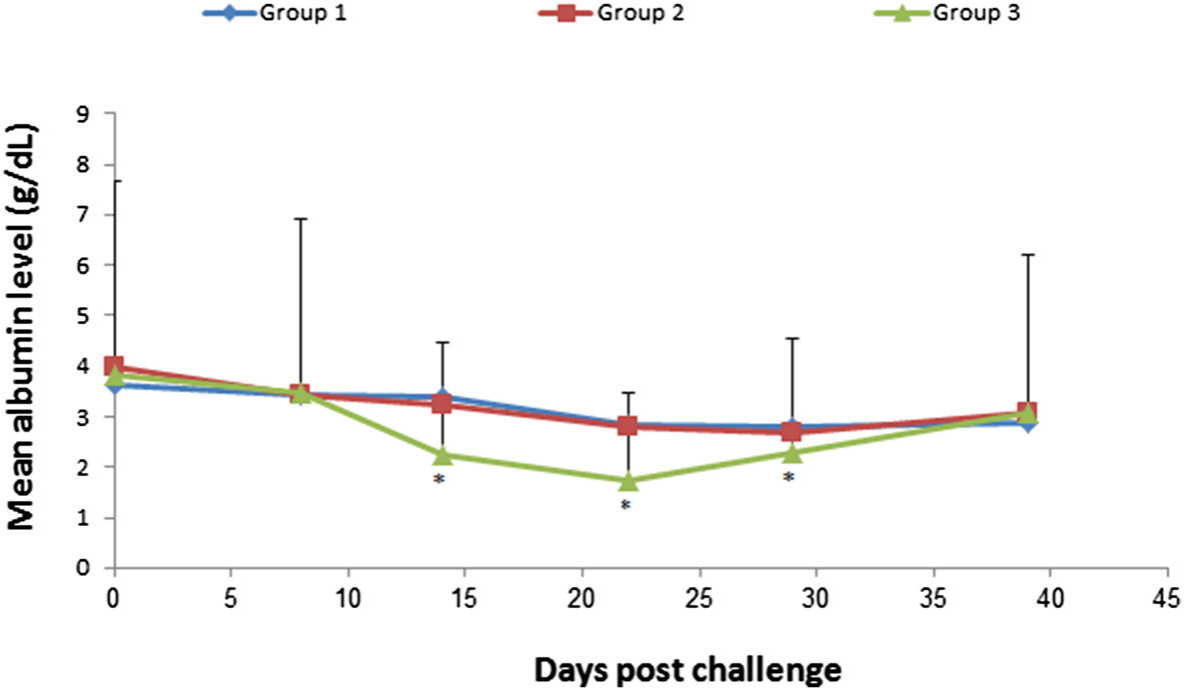
**Albumin kinetics in vaccinated and unvaccinated dog groups during the post-challenge phase.** Least square means sera level of albumin in vaccinated dogs (group 1 and 2) and control unvaccinated dogs (group 3) following challenge with a wild strain of *E. canis*. *Indicates significant difference between group 3 and group 1 (*p ≤* 0.02).

On day 14, mean albumin levels in group 3 were 1.3 g/dL lower than in group 1 (*p* < 0.001). Similarly, mean albumin levels were 1.3 g/dL lower (*p* = 0.002) and 0.72 g/dL lower (*p* = 0.02) on day 22 and 29 post-challenge, respectively (Figure 3). No differences between group 1 and group 2 were found.

#### TAC

##### Post-vaccination

Both groups 1 and 2 had significantly lower mean levels of TAC during the post-vaccination period. Compared to day 0, values on days 6, 13, 24, 31 and 38 were 0.15 mmol/L (*p* = 0.008), 0.24 mmol/L (*p* = 0.004), 0.2 mmol/L (*p* = 0.004), 0.27 mmol/L (*p* = 0.004) and 0.25 mmol/L (*p* = 0.009) lower, respectively.

##### Post-challenge

During most of the post-challenge phase, TAC levels in all groups were lower than those in the pre-challenge phase. On days 8, 14, 22 and 29 post-challenge, values were 0.14 mmol/L (*p* = 0.01), 0.18 mmol/L (*p* = 0.002), 0.16 mmol/L, (*p* = 0.005) and 0.14 mmol/L (*p* = 0.01) lower, respectively. No differences were detected between groups 1 and 2 during this period.

#### PON-1

##### Post-vaccination

PON-1 levels in group 2 were 0.79 IU/mL greater on day 6 post-vaccination (*p* = 0.02) and 0.8 IU/mL greater on day 31 post-vaccination (*p* = 0.03), compared to those in group 1. On day 31 post-vaccination, PON-1 values were 0.73 IU/mL lower than those found in the pre-vaccination period (*p* = 0.004).

##### Post-challenge

Following challenge, all groups showed a decrease in the mean level of PON-1 from day 14 until day 39 post-challenge (day 14, 0.87 IU/mL, *p* = 0.01; day 22, 1.51 IU/mL, *p* < 0.001; day 29, 1.19 IU/mL, *p* = 0.001; day 39, 0.97 IU/mL, *p* =0.005). No significant differences in PON-1 levels were recorded between all groups throughout the challenge phase.

### Correlations between APP levels, clinical and hematological parameters

Correlation matrices for each phase are provided in Additional file 2: Table S2, Additional file 3: Table S3 and Additional file 4: Table S4

#### Post-vaccination phase

##### Group 1

The rickettsial load was found to be positively correlated with the Hp levels (*r* = 0.66, *p* = 0.005), CRP levels (*r* = 0.76, *p* < 0.001), and SAA levels (*r* = 0.61, *p* = 0.001). SAA levels positively correlated with the dog’s body temperatures (*r* = 0.52, *p* = 0.010).

Negatively correlated variables in the post-vaccination phase included rickettsial load with albumin (*r* = −0.4, *p* = 0.054) and TAC levels (*r* = −0.4, *p* = 0.035), and CRP levels with number of thrombocytes (*r* = −0.65, *p* = 0.007).

##### Group 2

In group 2 dogs, which served as controls for group 1 during the post-vaccination phase, albumin and PON-1 levels (*r* = 0.57, *p* = 0.008), correlated positively.

#### Post-challenge phase

Since the results differed between the pre-treatment period i.e. the time period from the day of challenge to the beginning of treatment with doxycycline (day 0 to 19 post challenge) and the period thereafter (treatment period, day 19 to 39 post-challenge), these periods were analyzed separately.

##### Group 1

In the pre-treatment period, rickettsial load correlated positively with CRP and SAA levels (*r* = 0.87, *p* = 0.005; *r* = 0.74, *p* = 0.013, respectively), and albumin levels correlated negatively with body temperatures (*r* = −0.63, *p* = 0.034).

In the treatment period, the rickettsial load was correlated positively with the CRP level (*r* = 0.88, *p* = 0.003) and body temperature (*r* = 0.62*, p* = 0.041).

Negatively correlated variables during the post-treatment initiation period included the rickettsial load and thrombocyte numbers (*r* = −0.94*, p* < 0.001), and CRP levels and number of thrombocytes (*r* = −0.84*, p* = 0.003).

##### Group 2

Variables found to be positively correlated in the pre-treatment period included rickettsial load and temperature (*r* = 0.66*, p* = 0.018), CRP and SAA levels (*r* = 0.89*, p* = 0.001), albumin and PON-1 levels (*r* = 0.59*, p* = 0.043), and PON-1 and TAC levels (*r* = 0.97*, p* < 0.001). Negatively correlated variables during the pre-treatment period included the rickettsial load and albumin levels (*r* = −0.71*, p* = 0.009).

In the treatment period, rickettsial load correlated positively with both CRP and SAA levels (*r* = 0.72, *p* = 0.007; *r* = 0.77, *p* = 0.003, respectively), and also with body temperature (*r* = 0.61*, p* = 0.031). CRP levels correlated positively with body temperature (*r* = 0.68*, p* = 0.013).

Negatively correlated variables during the treatment period included the rickettsial load with PON-1 levels (*r* = −0.71*, p* = 0.008), thrombocyte numbers (*r* = −0.77*, p* = 0.003), and TAC levels (*r* = −0.74*, p* = 0.005); CRP levels and thrombocyte numbers (*r* = −0.73*, p* = 0.006), SAA and thrombocyte numbers (*r* = −0.67*, p* = 0.003), and PON-1 levels with body temperature (*r* = −0.79*, p* = 0.002).

##### Group 3

Variables found to be positively correlated in the pre-treatment period included: rickettsial load and CRP (*r* = 0.81*, p* = 0.004) and SAA levels (*r* = 0.82*, p* = 0.003); albumin level and thrombocyte numbers (*r* = 0.87*, p* = 0.002), and body temperature and CRP level (*r* = 0.57, *p* = 0.049).

Negatively correlated variables during the pre-treatment period included the rickettsial load and thrombocyte numbers (*r* = −0.92*, p* = 0.008), rickettsial load and albumin level (*r* = −0.66, *p* = 0.035), CRP levels and thrombocyte numbers (*r* = −0.93*, p* = 0.002), SAA levels and thrombocyte numbers (*r* = −0.94*, p* = 0.001), and PON-1 levels and body temperature (*r* = −0.67, *p* = 0.021).

During the treatment period, positive correlations were found between rickettsial load and CRP levels (*r* = 0.79*, p* = 0.010) as well as SAA level (*r* = 0.61*, p* = 0.056). In addition, Hp levels were correlated positively with the thrombocyte numbers (*r* = 0.7*, p* = 0.016).

Variables found to be negatively correlated during the treatment period included rickettsial load and body temperature (*r* = −0.76*, p* = 0.005), and SAA and body temperature (*r* = −0.88, *p* = 0.007).

## Discussion

Studies on the evaluation of innate inflammatory responses following vaccination trials are uncommon. Typically, parameters relating to the acquired immune responses such as antibody formation and lymphocyte proliferation are studied. In this study, the production of the APP, representing markers of innate inflammatory response, were shown to be considerably elevated or decreased, depending on their role as positive or negative APP, in challenged unvaccinated dogs, compared to challenged vaccinated dogs. Furthermore, the peak levels of various APP responses were not only smaller in vaccinated dogs but also appeared later on a time scale compared to challenged unvaccinated dogs. Changes in the concentrations of APP were noted also during the vaccination phase of the study as expected following vaccination with a live bacterial strain (group 1) and also following inoculation of uninfected DH-82 cells (group 2), however, these were of lower magnitude compared to most alterations in the challenged unvaccinated dogs. This provides important information on the dynamics of responses to vaccination with the attenuated *E. canis* strain, and the protection that it confers to vaccinated dogs in the perspective of the innate immune system responses. This study complements a previous study which focused on clinical, hematologic, serologic and bacterial load parameters and found up to 92 fold higher rickettsial load in unvaccinated challenged dogs compared to vaccinated dogs post-challenge, associated with severe disease versus no disease in the vaccinated dogs [14]. Due to the inoculation of dogs with blood from a naturally-infected dog presenting clinical *E. canis* infection, and although this blood was negative by PCR for *Babesia* spp. and *H. canis* and by blood smear microscopy for other parasites, the possibility that other pathogens were transmitted during inoculation cannot be ruled out. However, the inoculated vaccinated dogs did not develop disease with additional pathogens.

CRP which is the most studied APP in dogs and frequently found indicative as an inflammatory marker in dogs, was markedly elevated in unvaccinated dogs (group 3) post-challenge. It reached the peak level recorded in the study, which was 50 times higher than the pre-challenge level, 14 days post-challenge compared to considerably lower peak levels in the vaccinated groups, reached more than a week later, on day 22. Although CRP levels increased post-vaccination in the vaccinated group, they only reached much lower levels. A similar pattern with earlier and higher increases in APP levels in the control group post-challenge and with generally lower levels of APP during vaccination was also found for SAA, whose level was 94 times higher than pre-challenge on day 14 post-challenge and for Hp. An inverse response was found for the negative APP Albumin which decreased after challenge in the control group and with a considerably lower magnitude in the vaccinated groups. These findings are in agreement with the presentation of the clinical disease that became severe in the challenged control group and was unapparent in the vaccinated groups.

The strong positive correlation between the rickettsial load and positive APP such as CRP and SAA suggests that bacterial loads affected the production of these APP. Lower levels of these APP were associated with the low rickettsial load found in vaccinated dogs whereas considerably higher levels were demonstrated in challenged unvaccinated dogs with a high rickettsial load. Hence, CRP and SAA levels may serve as good indicators for the magnitude of infection with *E. canis* during the acute phase of the disease. Conversely, decreasing albumin levels were positively correlated with the decreasing thrombocyte counts during the challenge phase in the unvaccinated dogs. In addition, the albumin level was negatively correlated with the rickettsial load in the challenged unvaccinated dogs. These findings demonstrate that the negative acute phase protein, albumin, may serve as a diagnostic indicator for more severe disease whose level parallels changes in thrombocyte counts and *E. canis* bacterial loads in infected dogs.

Most positive APP correlated positively with other positive APP and negatively with negative APP and oxidative markers. These correlations were stronger in the unvaccinated challenged control dogs in comparison to the vaccinated dogs, as the magnitude of APP responses was generally considerably higher in the unvaccinated dogs that were not protected by the vaccine.

No major differences were noted between the APP response in groups 1 and 2 during the challenge phase. This was also in agreement with the clinical, hematologic and rickettsial load outcomes [14]. Therefore, from the standpoint of evaluating the response to vaccination as it is seen by APP production, there was no advantage to vaccinating twice versus vaccinating the dogs once prior to challenge with a wild strain of *E. canis*.

A study on APP in naturally infected dogs [3] which compared dogs with non-myelosuppressed ehrlichiosis to dogs with myelosuppressive disease, presumed to have chronic infection, and control beagles, also found elevations in CRP, SAA and Hp and decreased albumin levels. These findings are essentially similar to our findings from the experimental vaccination study, with regard to the APP which elevate in *E. canis* infection. Interestingly, a higher proportion of dogs with CRP, SAA and Hp increases were found in naturally infected myelosupressed dogs in comparison to non-myelosupressed dogs, indicating that probably as CME becomes chronic and the clinical presentation worsens, production of these APP is further increased. In contrast, albumin decrease in the naturally infected dogs was not different between myelospuressed and non-myelosupressed dogs [3]. Other studies in experimentally infected dogs with CME have documented increases in CRP, Hp, acid-glycoprotein, ceruloplasmin, transferrin, and decrease in albumin [4,5,25].

In a different canine vaccination and challenge study, canine parvovirus infection of vaccinated and unvaccinated control puppies resulted in a clinical disease with virus shedding in the feces in the control dogs and an increase in the levels of SAA and α-l acid glycoprotein (α-l AG), whereas vaccinated dogs did not develop a clinical disease and had lower levels of these APP [26]. These results are comparable to the results in our study on *E. canis* vaccination and challenge, although we evaluated a wider range of positive and negative APP.

Different panels of APP have been studied in natural and experimental infection with various canine vector-borne diseases other than CME including leishmaniosis caused by *Leishmania infantum* [27-31] and babesiosis with different species of *Babesia* [32-34]. These studies have generally been found helpful in evaluating the magnitude and severity of the disease as well as improvement in dog condition following effective treatment.

PON-1 and TAC are inflammatory and metabolic indicators that have been less recently utilized in veterinary medicine [23]. Our study is the first to evaluate these oxidative stress markers in *E. canis* infection. Both markers decreased during the vaccination and challenge phases, however they could not distinguish clearly between control dogs with severe disease and clinically normal, vaccinated dogs. Further evaluation of the usefulness of PON-1 and TAC in *E. canis* infection including the chronic disease stage may be warranted.

## Conclusions

Vaccination with an attenuated *E. canis* strain in an experimental CME infection resulted in considerably muted positive and negative APP responses compared to those found in challenged unvaccinated dogs, reflecting a milder innate inflammatory response conferred by protection of the vaccine. These milder responses correlated well with the absence of clinical disease and diminished rickettsial load found in the vaccinated dogs post-challenge.

## Additional files

Additional file 1: Table S1. APP and antioxidant values during the post-vaccination and post-challenge periods. Additional file 2: Table S2. Correlations among APP, antioxidant analytes and clinical parameters during the post-vaccinal phase. Additional file 3: Table S3. Correlations among APP, antioxidant analytes and clinical parameters during the pre-treatment phase following challenge. Additional file 4: Table S4. Correlations among APP, antioxidant analytes and clinical parameters during the treatment phase following challenge.
