# Supplementary material for: Comparison of the acute phase protein and antioxidant responses in dogs vaccinated against canine monocytic ehrlichiosis and naive-challenged dogs
Source: Parasit Vectors. 2015 Mar 23;8:175. doi: 10.1186/s13071-015-0798-1 (PMC4371631; doi:10.1186/s13071-015-0798-1)
Supplement: Additional file 2: Table S2. — Correlations among APP, antioxidant analytes and clinical parameters during the post-vaccinal phase. [file 13071_2015_798_MOESM2_ESM.docx]

**Additional file 2: Table S2**: Correlations among APP, antioxidant analytes and clinical parameters during the post-vaccinal phase.

Legend: 1= group 1 (vaccinated), 2= group 2 (control). Upper values in each box denote the Spearman correlation coefficient. Lower values denote significance (*p*). *p*<0.05 is considered significance (significant values marked in bold).

| **Thrombocytes** | | **Rickettsial load** | | **PON-1** | | **TAC** | | **Albumin** | | **SAA** | | **Haptoglobin** | | **CRP** | |  |
| --- | --- | --- | --- | --- | --- | --- | --- | --- | --- | --- | --- | --- | --- | --- | --- | --- |
| 2 | 1 | 2 | 1 | 2 | 1 | 2 | 1 | 2 | 1 | 2 | 1 | 2 | 1 | 2 | 1 |  |
|  |  |  |  |  |  |  |  |  |  |  |  |  |  | 0.37 0.094 | **0.74 <0.001** | **Haptoglobin** |
|  |  |  |  |  |  |  |  |  |  |  |  | -0.040 0.862 | **0.76 <0.001** | 0.38 0.085 | **0.55 0.007** | **SAA** |
|  |  |  |  |  |  |  |  |  |  | -0.16 0.481 | **-0.41 0.048** | 0.23 0.301 | **-0.41 0.047** | -0.35 0.118 | -0.39 0.066 | **Albumin** |
|  |  |  |  |  |  |  |  | 0.039 0.872 | **0.52 0.011** | **-0.016 0.946** | -0.073 0.745 | **-0.64 0.003** | -0.18 0.422 | -0.41 0.080 | -0.29 0.201 | **TAC** |
|  |  |  |  |  |  | **0.47 0.042** | **0.49 0.010** | **0.57 0.008** | 0.23 0.281 | -0.023 0.923 | -0.041 0.852 | 0.054 0.819 | 0.15 0.482 | -0.25 0.277 | -0.012 0.957 | **PON-1** |
|  |  |  |  |  | -0.25 0.231 |  | **-0.45 0.035** |  | **-0.40 0.054** |  | **0.61 0.001** |  | **0.66 0.005** |  | **0.76 <0.001** | **Rickettsial load** |
|  |  |  | **-0.76 0.005** | 0.036 0.886 | **0.36 0.165** | 0.098 0.708 | **0.55 0.025** | -0.079 0.974 | 0.37 0.153 | 0.15 0.522 | -0.46 0.066 | -0.17 0.472 | -0.45 0.008 | **-0.049 0.840** | **-0.65 0.007** | **Thrombocytes** |
| 0.23 0.284 | -0.11 0.664 |  | 0.27 0.187 | -0.055 0.814 | -0.30 0.153 | 0.016 0.945 | -0.19 0.381 | 0.18 0.422 | **-0.19 0.367** | 0.15 0.512 | **0.52 0.010** | -0.086 0.709 | 0.35 0.099 | 0.10 0.649 | 0.098 0.663 | **Temperature** |
