# Supplementary material for: Comparison of the acute phase protein and antioxidant responses in dogs vaccinated against canine monocytic ehrlichiosis and naive-challenged dogs
Source: Parasit Vectors. 2015 Mar 23;8:175. doi: 10.1186/s13071-015-0798-1 (PMC4371631; doi:10.1186/s13071-015-0798-1)
Supplement: Additional file 3: Table S3. — Correlations among APP, antioxidant analytes and clinical parameters during the pre-treatment phase following challenge. [file 13071_2015_798_MOESM3_ESM.docx]

**Additional file 3: Table S3**: Correlations among APP, antioxidant analytes and clinical parameters during the pre-treatment phase following challenge.

Legend: 1= group 1 (vaccinated twice), 2= group 2 (vaccinated once), 3= group 3 (control). The upper values in each box denote the Spearman correlation coefficient. The lower values denote significance (*p*). *p*<0.05 is considered significant (significant values marked in bold).

| **Thrombocytes** | | | **Rickettsial load** | | | **PON-1** | | | **TAC** | | | **Albumin** | | | **SAA** | | | **Haptoglobin** | | | | **CRP** | | | |  | |
| --- | --- | --- | --- | --- | --- | --- | --- | --- | --- | --- | --- | --- | --- | --- | --- | --- | --- | --- | --- | --- | --- | --- | --- | --- | --- | --- | --- |
| 3 | 2 | 1 | 3 | 2 | 1 | 3 | 2 | 1 | 3 | 2 | 1 | 3 | 2 | 1 | 3 | 2 | 1 | | 3 | 2 | 1 | | 3 | 2 | 1 | |  |
|  |  |  |  |  |  |  |  |  |  |  |  |  |  |  |  |  |  | |  |  |  | | **0.71 0.008** | 0.1 0.7 | -0.14 0.6 | | **Haptoglobin** |
|  |  |  |  |  |  |  |  |  |  |  |  |  |  |  |  |  |  | | **0.55 0.05** | -0.1 0.7 | 0.22 0.5 | | **0.92 <0.001** | **0.89 0.001** | **0.78 0.006** | | **SAA** |
|  |  |  |  |  |  |  |  |  |  |  |  |  |  |  | **-0.85 0.003** | **-0.76 0.006** | -0.26 0.4 | | **-0.65 0.02** | 0.17 0.5 | -0.10 0.7 | | **-0.82 0.001** | **-0.78 0.007** | -0.18 0.5 | | **Albumin** |
|  |  |  |  |  |  |  |  |  |  |  |  | 0.55 0.06 | 0.57 0.06 | **0.67 0.02** | -0.44 0.1 | **-0.6**9 **0.02** | -0.51 0.1 | | **-0.67** **0.01** | -0.23 0.4 | -0.28 0.4 | | **-0.58 0.04** | -0.45 0.21 | -0.41 0.2 | | **TAC** |
|  |  |  |  |  |  |  |  |  | 0.53 0.08 | **0.97 <0.001** | 0.041 0.9 | **0.81 0.002** | **0.59 0.04** | 0.34 0.2 | **-0.63 0.03** | **-0.66 0.02** | 0.031 0.9 | | **-0.70 0.01** | -0.01 0.9 | 0.57 0.06 | | **-0**.**68 0.01** | -0.48 0.1 | -0.45 0.1 | | **PON-1** |
|  |  |  |  |  |  | -0.42 0.2 | -0.42 0.1 | -0.51 0.1 | -0.38 0.2 | -0.41 0.2 | -0.26 0.4 | **-0.66 0.03** | **-0.71 0.009** | -0.13 0.6 | **0.82 0.003** | 0.40 0.2 | **0.74 0.01** | | 0.34 0.3 | 0.23 0.4 | -0.16 0.6 | | **0.81 0.004** | 0.60 0.06 | **0.87 0.005** | | **Rickettsial load** |
|  |  |  | **-0.92 0.008** | -0.61 0.08 | -0.34 0.3 | -0.42 0.2 | -0.42 0.1 | -0.51 0.1 | 0.52 0.1 | -0.025 0.9 | 0.22 0.5 | **0.87 0.002** | 0.50 0.1 | -0.22 0.5 | **-0.94 0.001** | 0.25 0.5 | -0.26 0.5 | | -0.52 0.14 | 0.19 0.6 | -**0.86 0.008** | | -**0.93 0.002** | -0.41 0.3 | -0.34 0.3 | | **Thrombocytes** |
| -0.180.63 | -0.10 0.7 | 0.013 0.9 | 0.51 0.1 | **0.66 0.01** | 0.22 0.5 | 0.53 0.1 | 0.17 0.5 | 0.037 0.9 | -0.039 0.9 | -0.085 0.8 | -0.55 0.07 | -0.46 0.1 | 0.22 0.4 | **-0.63 0.03** | 0.48 0.1 | 0.066 0.8 | 0.58 0.07 | | -0.51 0.08 | 0.51 0.08 | 0.33 0.3 | | **0.57 0.04** | 0.30 0.3 | -0.34 0.3 | | **Temperature** |
