# Supplementary material for: Comparison of the acute phase protein and antioxidant responses in dogs vaccinated against canine monocytic ehrlichiosis and naive-challenged dogs
Source: Parasit Vectors. 2015 Mar 23;8:175. doi: 10.1186/s13071-015-0798-1 (PMC4371631; doi:10.1186/s13071-015-0798-1)
Supplement: Additional file 4: Table S4. — Correlations among APP, antioxidant analytes and clinical parameters during the treatment phase following challenge. [file 13071_2015_798_MOESM4_ESM.doc]

**Additional file 4: Table S4**: Correlations among APP, antioxidant analytes and clinical parameters during the treatment phase following challenge.

Legend: 1= group 1 (twice vaccinated), 2= group 2 (once vaccinated), 3= group 3 (control).

The upper values in each box denote the Spearman correlation coefficient. The lower values denote significance (*p*). *p*<0.05 is considered significant (significant values marked in bold).

| **Thrombocytes** | | | **Rickettsial load** | | | **PON-1** | | | **TAC** | | | **Albumin** | | | **SAA** | | | **Haptoglobin** | | | **CRP** | | | |  |
| --- | --- | --- | --- | --- | --- | --- | --- | --- | --- | --- | --- | --- | --- | --- | --- | --- | --- | --- | --- | --- | --- | --- | --- | --- | --- |
| 3 | 2 | 1 | 3 | 2 | 1 | 3 | 2 | 1 | 3 | 2 | 1 | 3 | 2 | 1 | 3 | 2 | 1 | 3 | 2 | 1 | | 3 | 2 | 1 |  |
|  |  |  |  |  |  |  |  |  |  |  |  |  |  |  |  |  |  |  |  |  | | -0.33 0.3 | **0.81 0.001** | 0.29 0.3 | **Haptoglobin** |
|  |  |  |  |  |  |  |  |  |  |  |  |  |  |  |  |  |  | -0.44 0.1 | 0.41 0.1 | **0.68 0.02** | | **0.69 0.02** | **0.60 0.03** | 0.52 0.1 | **SAA** |
|  |  |  |  |  |  |  |  |  |  |  |  |  |  |  | **-0.93 <0.001** | -0.31 0.3 | -0.079 0.8 | 0.18 0.5 | -0.18 0.5 | 0.068 0.8 | | **-0.72 0.02** | -0.49 0.1 | -0.12 0.71 | **Albumin** |
|  |  |  |  |  |  |  |  |  |  |  |  | **0.59 0.05** | **0.82 0.01** | 0.55 0.07 | -0.16 0.6 | **-0.67 0.01** | 0.090 0.8 | -0.23 0.4 | -0.097 0.7 | 0.072 0.8 | | -0.32 0.3 | -0.44 0.1 | -0.46 0.1 | **TAC** |
|  |  |  |  |  |  |  |  |  | 0.46 0.1 | **0.61 0.03** | **0.71 0.01** | **0.73 0.01** | **0.81 0.002** | **0.80 0.002** | **-0.65 0.04** | -0.42 0.1 | -0.34 0.3 | 0.57 0.06 | -0.24 0.4 | 0.041 0.9 | | **-0.84 0.001** | **-0.67 0.01** | **-0.58 0.05** | **PON-1** |
|  |  |  |  |  |  | -0.54 0.1 | **-0.71 0.008** | -0.54 0.08 | -0.094 0.7 | **-0.74 0.05** | -0.39 0.2 | -0.37 0.2 | **-0.54 0.008** | -0.23 0.4 | **0.61 0.05** | **0.77 0.003** | 0.35 0.3 | -0.18 0.5 | 0.32 0.3 | 0.34 0.2 | | **0.79 0.01** | **0.72 0.007** | **0.88 0.003** | **Rickettsial load** |
|  |  |  | -0.40 0.2 | **-0.77 0.003** | **-0.94 <0.001** | **0.67 0.03** | 0.47 0.1 | **0.65 0.05** | 0.054 0.8 | 0.53 0.07 | **0.75 0.01** | 0.39 **0.2** | 0.48 0.1 | 0.30 0.4 | -0.44 0**.2** | **-0.77 0.003** | -0.33 0.4 | **0.70 0.01** | -0.53 0.07 | -0.33 0.3 | | -0.62 0.07 | **-0.73 0.006** | **-0.84** **0.003** | **Thrombocytes** |
| 0.46 0.1 | -0.39 0.2 | -0.61 0.07 | **-0.076 0.05** | **0.61 0.03** | **0.62 0.04** | 0.49 0.1 | **-0.79 0.002** | -0.027 0.9 | 0.37 0.2 | -0.35 0.2 | 0.27 0.4 | 0.54 0.08 | -0.55 0.07 | **0.2**2 0**.**5 | **-0.73 0.009** | 0.32 0.3 | 0.42 0.2 | 0.21 0.4 | 0.24 0.4 | 0.45 0.1 | | **-0.88 0.007** | **0.68 0.01** | 0.53 0.08 | **Temperature** |
